# In the Pursuit of Metabolic Markers of Systemic Sclerosis—Plasma Adiponectin and Omentin-1 in Monitoring the Course of the Disease

**DOI:** 10.3390/ijms24129988

**Published:** 2023-06-10

**Authors:** Klaudia Dopytalska, Małgorzata Kalisz, Anna Litwiniuk, Irena Walecka, Wojciech Bik, Agnieszka Baranowska-Bik

**Affiliations:** 1 Department of Dermatology, Centre of Postgraduate Medical Education, 01-813 Warsaw, Poland; 2 Department of Dermatology, The National Institute of Medicine of the Ministry of Interior and Administration, 02-507 Warsaw, Poland; 3 Department of Neuroendocrinology, Centre of Postgraduate Medical Education, 01-813 Warsaw, Polandwojciech.bik@cmkp.edu.pl (W.B.); 4 Department of Endocrinology, Centre of Postgraduate Medical Education, 01-809 Warsaw, Poland

**Keywords:** systemic sclerosis, insulin resistance, omentin-1, adiponectin

## Abstract

Systemic sclerosis (SSc) is a connective tissue disease leading to cutaneous and visceral fibrosis. Pathological features of SSc include immune dysregulation, vasculopathy, and impaired angiogenesis. Adipokines act as cytokines and hormones and are involved in various pathological processes, including metabolic disorders, inflammation, vasculopathy, and fibrosis. This study aimed to determine the level of omentin-1 and adiponectin to evaluate their potential role in the pathogenesis of SSc. We assessed serum omentin-1 and adiponectin as well as metabolic parameters in 58 patients with SSc and 30 healthy controls. The follow-up was performed in SSc individuals. Omentin-1 levels were significantly higher in SSc individuals as compared to the controls. In post-hoc analysis, omentin-1 was higher in the group with disease duration ≥7 years than in the control group. A positive correlation was noted between disease duration and both adipokines and increased with longer disease duration. However, there were no correlations between selected adipokines and metabolic parameters. Enhanced omentin-1 levels and higher levels of omentin-1 in patients with longer disease duration may suggest that omentin-1 is involved in the pathomechanisms of SSc as its concentrations are not directly related to BMI, age, and insulin resistance.

## 1. Introduction

Systemic sclerosis (SSc) is a chronic and progressive connective tissue disease leading to skin, blood vessels, and visceral organ fibrosis. The characteristic clinical features include skin sclerosis, lung fibrosis, and gastrointestinal, cardiovascular, renal, and osteoarticular disorders. Depending on the clinical features, diffuse cutaneous (dc) and limited cutaneous (lc) SSc are distinguished [1,2,3,4]. The etiopathogenesis of SSc has not been fully understood, but autoimmune processes, vascular endothelium dysfunction, and excessive synthesis of collagen leading to tissue fibrosis play a crucial role in the development of the disease [5,6,7].

Adipose tissue is a source of biologically active compounds. Cells (primarily macrophages) located in adipose tissue synthesize and secrete increased amounts of pro-inflammatory factors (e.g., IL-1, IL-6, tumor necrosis factor α—TNFα). In addition, adiposetissue has immunomodulatory effects [8,9]. There are reports that obesity worsens the course of rheumatoid arthritis, systemic lupus erythematosus, and psoriatic arthritis [8]. Although the majority of patients with systemic sclerosis have a normal Body Mass Index (BMI), studies have indicated unfavorable changes in the concentrations of some adipokines in this group of patients [10,11,12].

Insulin resistance (IR) is a pathological condition of impaired biological response of tissues to insulin, despite normal or elevated insulin concentration in the blood. As a result of metabolic disorders associated with IR, chronic low-grade inflammation is enhanced [13]. Studies have shown a correlation between IR and pro-inflammatory cytokines, in particular TNF-α [14]. Interestingly, IR incidence has been higher in patients with systemic connective tissue diseases, such as rheumatoid arthritis and systemic lupus erythematosus [15]. Furthermore, despite BMI being within a normal range in the majority of patients with systemic sclerosis, IR is observed in this group, primarily in those with ulceration of the fingers [16].

Adipokines act as cytokines and hormones. They are involved in the regulation of energy metabolism, food intake, and numerous physiological and pathophysiological processes [17,18]. Adipokines are capable of activating immune cells leading to the accumulation of inflammatory cells and affecting T-cell differentiation, and surprisingly showing both pro-inflammatory and anti-inflammatory effects [19,20,21]. Studies have shown that a disturbed adipokine profile could be potentially involved in various pathological processes in cardiovascular, metabolic, rheumatic, and autoimmune disorders, including SSc [20,22,23,24,25,26].

Omentin-1 is a 313 amino-acids adipokine. The biological role of omentin-1 in the physiological and pathophysiological processes is still not fully understood [27,28]. It is synthesized predominantly in visceral adipose tissue (VAT). Two isoforms of this protein are distinguished: Omentin-1 (circulating isoform) and omentin-2 secreted into the intestine lumen [28]. It has been presumed that omentin-1 shows vasoprotective and vasodilatory effects by inhibiting cyclooxygenase-2 (COX-2) and reactive oxygen species (ROS) in endothelial cells and stimulating nitric oxide (NO) synthesis. Moreover, omentin-1 suppresses the expression of adhesions molecules, including intercellular adhesion molecule-1 (ICAM-1) and vascular adhesion molecule-1 (VCAM-1) by the impact on the nuclear factor kappa-light-chain-enhancer of activated B cells (NF-κB) signaling pathway and inhibition of monocytes adhesion. Omentin-1 also affects the 5′ AMP-activated protein kinase (AMPK) pathway. Therefore, it may show anti-inflammatory properties [28,29,30]. Recently, the potential role of omentin-1 in autoimmune disorders, including psoriasis, has been suggested [31,32]. The role of omentin-1 in the pathogenesis of systemic sclerosis is still under study.

However, it is suggested that the loss of the protective effect of omentin-1 on blood vessels may be involved in pathomechanisms leading to vascular damage in the course of systemic sclerosis [33]. Ometin-1 activates the endothelial nitric oxide synthase (eNOS) signaling pathway and inhibits oxidative stress, leading to increased survival and proliferation of endothelial cells and the promotion of revascularization [34]. In the early stages of systemic sclerosis, characterized by excessive activation of the vascular endothelium, decreased omentin-1 levels may contribute to the loss of neovascularization and abnormal vascular remodeling leading to vascular damage and vasculopathy [33]. 

Adiponectin is mainly synthesized by adipocytes. Other cells have also been shown to secrete adiponectin, including macrophages, leukocytes, fibroblasts, and endothelial cells [35,36]. This adipokine is a polypeptide of 244 amino acids and its molecular weight is 30 kDa [17,36]. Three forms of adiponectin are formed: Low molecular weight (LMW) fraction, medium molecular weight (MMW) fraction, and high molecular weight (HMW) fraction. Adiponectin is also found in the globular form [35,37]. The biological activity of adiponectin is mainly due to the high-molecular fraction, HMW [36,38], that has a stronger metabolic, vascularprotective, and cardioprotective effect compared to total adiponectin [39,40]. 

The concentration of adiponectin is reduced in obesity and shows a negative correlation with the amount of adipose tissue, BMI, and indicators of insulin resistance. Adiponectin synthesis increases with weight loss and is inhibited by pro-inflammatory molecules (TNF-α, IL-6) [17]. Adiponectin enhances insulin sensitivity and has a beneficial effect on the lipid profile [36], as well as shows anti-inflammatory and anti-atherosclerotic effects. It directly affects the vascular endothelium showing a vasoprotective effect. It reduces the accumulation of lipids in the vessels, inhibits the transformation of macrophages into foam cells and the proliferation of vascular smooth muscle cells, and reduces the production of pro-inflammatory cytokines (including TNF-α), as well as adhesion molecules. Adiponectin also stimulates the production of NO, promotes repair mechanisms of endothelial cells, and stimulates angiogenesis [35,41,42,43].

Data on the role of adiponectin in systemic sclerosis are limited. Reduced levels of adiponectin in patients with SSc have been shown [44,45]. Significantly lower concentrations of adiponectin were also observed in patients with diffuse cutaneous systemic sclerosis compared to those with the limited form [44,46]. In addition, it has been found that the concentration of adiponectin changes with the duration of the disease, as a lower concentration of adiponectin occurs in patients in the early stage of the disease (≤5 years) when the process of fibrogenesis is the most intense. Most of the published data suggest that decreased adiponectin levels may be associated with skin fibrosis [25,44,46,47,48]. The results also indicate a negative correlation between the level of adiponectin and the severity of skin fibrosis according to the modified Rodnan skin score in patients with diffuse cutaneous SSc [46,47]. 

The results obtained so far do not fully explain the role of omentin-1 and adiponectin in systemic sclerosis. Therefore, this study aimed to determine the level of selected adipokines, omentin-1, and adiponectin, concerning the metabolic status and to evaluate their potential role in the pathological processes in SSc. 

## 2. Results

In the group of patients with systemic sclerosis, the BMI index had a median of 23.35, and in the control group the median was 24.71. There were no significant differences between the two groups for BMI as well as for other anthropometric parameters, body composition, and HOMA-IR. Biochemical parameters between the SSc and control groups markedly differed for the triglyceride level and C-reactive protein (CRP). Triglycerides were the highest in the SSc group (*p* = 0.024). The CRP level was also higher in patients with SSc compared with the control group (*p* = 0.029) (Table 1).

The comparison of adipokine levels revealed a significant difference in the concentration of omentin-1 between the SSc group and the control group, with the higher level in the SSc group (*p* = 0.030). For total adiponectin and HMW adiponectin, we did not reveal statistical differences between both analyzed groups (Table 2). 

In addition, we assessed correlations between adipokines and age and adipokines and CRP. No correlations were observed (Table 3).

In the whole SSc group, the diffuse form of the disease was present in 40 cases (69% of all SSc subjects), while the limited form was diagnosed in 18 individuals (31%). When anthropometric and biochemical parameters and adipokines were analyzed between the subgroups of SSc and the controls (diffuse SSc vs. limited SSc, diffuse SSc vs. controls and limited SSc vs. controls) the significant differences were observed between limited SSc and the controls in terms of age and triglycerides (*p* = 0.04 and *p* = 0.034, respectively (Table 4).

When the SSc subjects were divided according to the disease duration with a cutting point of 7 years in the group of shorter disease duration (*n* = 26) the diffuse form was found in 19 individuals, while the limited form was observed in 7 cases. When the group of patients with longer disease duration (*n* = 32) was analyzed, it was revealed that the diffuse form was found in 21 subjects, and the limited form was seen in 11 cases. 

Furthermore, amongst the SSc group, pulmonary involvement confirmed with computed tomography was present in 33 patients (56.9%), arthritis was observed in 26 (44.8%) individuals and gastrointestinal tract complications were seen in 32 patients (55.2%). In addition, 1 patient had a scleroderma renal crisis in anamnesis.

The assessment of the correlation of pulmonary involvement with adipokines revealed no correlation in all studied models (SSc with pulmonary involvement vs. SSc without pulmonary involvement, SSc with pulmonary involvement vs. controls, and SSc without pulmonary involvement vs. controls). 

The antibodies pattern in the SSc patients showed that Anti-Scl70 was found in 36 (62.1%) subjects. The measured antibodies pattern is presented in Table 5. 

The SSc group was divided according to the duration of the disease using the median value (7 years). A group of patients with a disease duration of fewer than 7 years (26 individuals) and a group of individuals with a disease duration of 7 years or more (32 individuals) were created. Then, two groups were compared with the control group in terms of individual anthropometric, biochemical, and adipokine parameters. There was no significant difference in age and BMI values between the three analyzed subgroups. Only omentin-1 significantly differed between the analyzed groups: Omentin-1 (*p* = 0.019). Post-hoc analysis showed that omentin-1 was higher in the group with disease duration ≥7 years than in the control group. For total adiponectin and HMW adiponectin, no significant differences were found between subgroups (Table 6).

The correlation between the modified Rodnan skin score and adipokines was also assessed, and the marked correlation and HMW-adiponectin were revealed (rho −0.27, *p* = 0.048) (Table 7).

**Figure 1:**
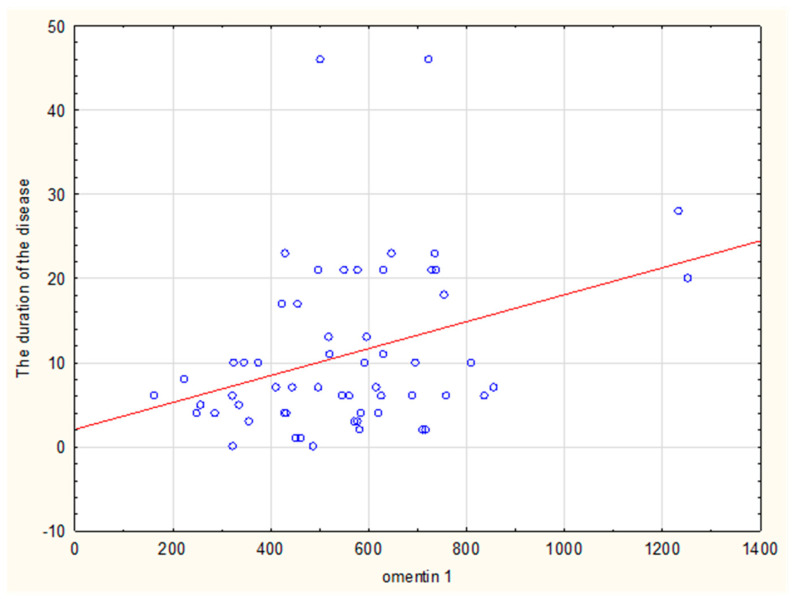
Correlation of omentin-1 and the duration of the disease.

**Figure 2:**
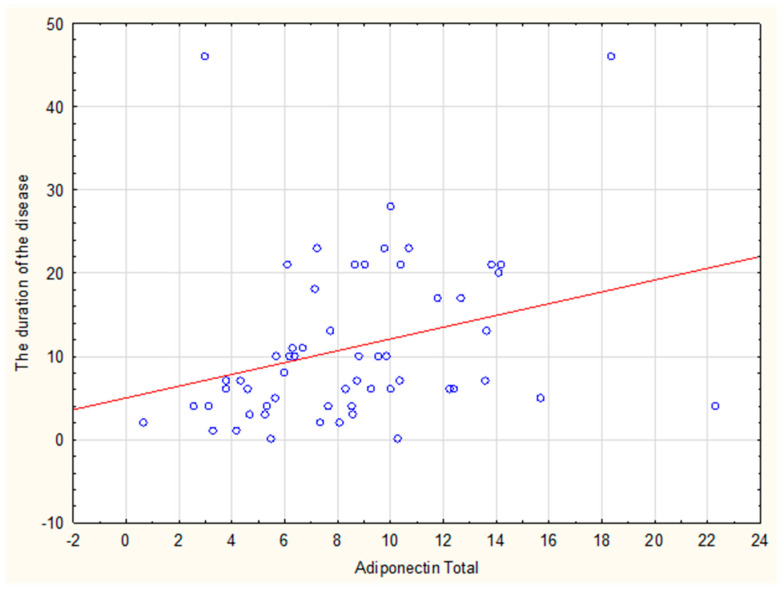
Correlation of adiponectin (total) and the duration of the disease.

A significant correlation was confirmed between the duration of the disease and the level of omentin-1 (rho = 0.32) and adiponectin (rho = 0.38) (Figure 1 and Figure 2, respectively).

However, omentin-1, adiponectin, and HMW adiponectin did not change between measurements during the 9-month follow-up (Table 8).

The frequency of comorbidities between the groups was also analyzed. Hypertension was significantly more frequent in the SSc group (38%) than in the control group (13%), RR = 2.84, CI95 [1.08; 7.50], *p* = 0.025. The incidence of other diseases (type 2 diabetes, ischemic heart disease, myocardial infarction) did not differ significantly between the two groups (Table 6). An additional statistical analysis showed no influence of the presence of arterial hypertension on the obtained results (Table 9).

## 3. Discussion

Studies on the occurrence of metabolic disorders, insulin resistance, and body composition analysis in patients with systemic sclerosis are scarce. Interestingly, despite BMI within the normal range, which is present in the majority of patients with systemic sclerosis, results of some studies indicated disturbed body composition in this group of patients [49]. In addition, the research by Park and co-workers demonstrated a statistically higher incidence of insulin resistance in patients with SSc as compared to the control group, even though significantly lower BMI in the group of patients suffering from SSc was seen [16]. It has been suggested that factors contributing to weight loss in patients with systemic sclerosis include malnutrition and muscle atrophy [50]. In addition, malfunction of internal organs, in particular the gastrointestinal tract, in the course of systemic sclerosis may lead to appetite loss and, consequently, reduced food intake and changes in body composition and structure. It is worth noticing that gastrointestinal symptoms in SSc patients are a risk factor for malabsorption and malnutrition, which lead not only to weight loss but also to a worse prognosis [10].

There is also a suggestion that IR is related to systemic connective tissue diseases. A higher incidence of IR has been demonstrated in patients with rheumatoid arthritis as well as those with systemic lupus erythematosus when compared to the controls [15,51]. Moreover, the study of Park et al. revealed a higher HOMA-IR index in patients with systemic sclerosis in comparison with the control group. In addition, this study also showed a positive correlation between HOMA-IR values and the presence of finger erosions. The authors suggested a potential role of insulin resistance in the pathogenesis of vascular abnormalities leading to erosion of the fingertips in SSc [16].

In contrast to the above-presented findings, in the present study, we did not find any significant differences in insulin and HOMA-IR in subjects with systemic sclerosis compared to their healthy counterparts. Furthermore, these parameters did not vary between patients with limited and diffuse cutaneous SSc. However, it should be highlighted that the median HOMA-IR showed a non-significant trend towards higher values in individuals with scleroderma. Finally, our results revealed that there was no relationship between tested adipokines and both insulin and the HOMA -IR index.

Studies on SSc pathogenesis have also indicated the potential involvement of adipokines in the pathogenesis of systemic sclerosis. However, the results of scientific research published so far are often inconclusive, and the role of these molecules in SSc is not fully understood [25].

The studies published so far have shown a reduced concentration of adiponectin in patients with diffuse cutaneous SSc compared to the control group and, additionally, in patients at an early stage of the disease when the fibrosis process is most intense [44,45,46,48]. A negative correlation between the level of adiponectin and the severity of skin fibrosis according to the modified Rodnan skin score in patients with dcSSc was also observed [46,48]. In a study by the team of Neumann, reduced expression of adiponectin in the lung tissue of individuals with pulmonary fibrosis in the course of SSc was demonstrated. Moreover, decreased expression of adiponectin was observed in the gastric tissue of these patients [52].

Contrary to published studies, we found no significant difference in adiponectin and HMW adiponectin levels in patients with systemic sclerosis compared to the control group. During the 9-month observation, no changes in the concentration of this adipokine were noticed. However, a negative correlation between the concentration of adiponectin and the long-time duration of the disease was found in our group under the study. Moreover, our observation of the negative correlation of the modified Rodnan skin score with the level of HMW adiponectin is noteworthy. This finding is in concordance with data from the literature suggesting that reduced adiponectin levels may be associated with skin fibrosis [25,46,47,48]. It is also speculated that lower levels of adiponectin in the blood serum in conjunction with the reduced activity of peroxisome proliferator-activated receptor γ (PPARγ) may cause the activation and progression of skin fibrosis, especially in the early stages of SSc [47]. Furthermore, the study by Arakawa et al. showed reduced serum adiponectin concentration as well as decreased adiponectin mRNA levels in the skin of individuals with diffuse cutaneous systemic sclerosis. Decreased levels of adiponectin were associated with more advanced skin sclerosis and a higher incidence of pulmonary fibrosis, suggesting that adiponectin concentration may be a useful marker for assessing fibrosis in the course of SSc [48]. However, in our study, there were no correlations between pulmonary involvement and adipokine concentration. Nevertheless, it has been assumed that the antifibrotic effect of adiponectin may be associated with the induction of 5′ AMP-activated protein kinase (AMPK) and the effect on PPARγ activity [53]. It has also been observed that the treatment of systemic sclerosis may affect the concentration of adiponectin. Noticeably, a significant increase in the concentration of adiponectin during the treatment of systemic sclerosis with epoprostenol was shown, and this phenomenon may have a beneficial effect by inhibiting the progression of fibrosis in the course of the disease [54]. Unfortunately, due to the complex regiment of the rheological therapy of our patients, there was no possibility of evaluating the correlation between this kind of treatment and adipokines.

So far, to our knowledge, only one study evaluated the concentration and influence of omentin-1 on the course of SSc. The authors showed no differences in the concentration of omentin-1 in the sera of patients with systemic sclerosis compared to the control group. However, lower concentrations were observed in individuals with diffuse cutaneous systemic sclerosis compared to its limited form and in SSC patients with shorter disease duration (≤5 years) compared to controls [33].

Our study revealed a significantly higher concentration of omentin-1 in patients with systemic sclerosis compared to the control group. Higher levels of this adipokine in patients with SSc were not directly related to BMI, patients’ age, and insulin resistance indices (HOMA-IR, fasting insulin levels), which were comparable in the SSc and control groups. In addition, a significant correlation was confirmed between the duration of the disease and the level of omentin-1 (rho = 0.32). Moreover, post-hoc analysis showed that omentin-1 was higher in the group with disease duration ≥7 years than in the control group. However, we are aware of the influence of small study groups on our findings. Nevertheless, our results are in concordance with a study by Miura et al., who also showed a similar correlation in patients with dSSc [33].

Omentin-1 has vasoprotective, vasodilating, and anti-inflammatory properties. It could be speculated that the enhanced omentin-1 serum concentration observed in our study might serve as a compensatory mechanism for vascular dysfunction associated with pathological processes in the course of SSc. The study by Miuro et al. showed elevated omentin-1 levels in SSc patients with increased right ventricle systolic pressure RVSP, which may be associated with a loss of response to the vasodilating effect of omentin-1 caused by eNOS suppression in epigenetic mechanisms or a compensatory increase in omentin-1 levels. These authors suggest that the level of omentin-1 may be a useful marker reflecting the advancement of pulmonary vascular involvement leading to the development of pulmonary hypertension as a result of a compensatory enhancement in the concentration of this adipokine caused by an increase in pulmonary artery pressure [33]. Taking into account the results obtained in our study as well as in the study by Miura et al., it could be hypothesized that omentin-1 may be involved in the process of vasculopathy in systemic sclerosis, especially in the early stages of the disease and additionally, in the development of pulmonary hypertension. However, further intensive research should be performed on a larger cohort of patients to evaluate the exact role of omentin-1.

## 4. Materials and Methods

### 4.1. The Patients with Systemic Sclerosis

The study group comprised 58 patients, including 46 women (79.3%) and 12 men (20.7%). The mean age was 54.38 ± 13.42 years (between 33 to 75 years). The study included individuals with systemic sclerosis that was diagnosed based on the criteria of the European League Against Rheumatism (EULAR) (2013) and the American College of Rheumatology (ACR) [55]. The disease duration from the diagnosis of SSc ranged from 3 months to 46 years (median 7 years). Forty (69%) patients were diagnosed with diffuse cutaneous sclerosis, and 18 (31%) with limited systemic sclerosis. 

Thirty-two patients had immunosuppressive therapy (mycophenolate mofetil *n* = 26. methotrexate *n* = 5, cyclophosphamide *n* = 1). Rheological treatment included different combinations, times of use, and doses of sildenafil, sulodexide, and alprostadil.

Patients were recruited from the Department of Dermatology, Centre of Postgraduate Medical Education located in The National Institute of Medicine of The Ministry of Interior and Administration in Warsaw, Poland.

### 4.2. The Control Group

The control group consisted of 30 individuals, 25 women (83.3%) and 5 men (16.7%). The mean age in the control group was 49.57 ± 10.68 years. The control group comprised patients from the Department of Dermatology, Centre of Postgraduate Medical Education, and The National Institute of Medicine of The Ministry of Interior and Administration in Warsaw, Poland who were without any signs of systemic sclerosis and in whom autoimmune skin diseases were excluded. No significant differences were found for BMI, anthropometric parameters, and body composition in patients with systemic sclerosis and the control group (Table 1). The comorbidities comparison of both groups is presented in Table 9.

### 4.3. Criteria for Exclusion from the Study and Informed Consent

Criteria for exclusion from the study.

- Lack of informed patient consent.
- Age under 18.
- Severe liver and kidney failure.
- Advanced heart failure (NYHA grade III and IV).
- Active neoplastic disease and no proven remission of neoplastic disease.
- Acute infectious disease.
- Uncontrolled hypothyroidism and other severe endocrinopathies, including adrenal insufficiency, Cushing’s syndrome, hyperthyroidism.
- Alcoholism, drug addiction.

Each participant was informed about the purpose of the study, and written consent was obtained at the beginning. The research project protocol was approved by the Bioethical Committee of the Central Clinical Hospital of the Ministry of Interior and Administration in Warsaw (resolution 2/2016 of 13 January 2016). The study was in accordance with the Declaration of Helsinki.

### 4.4. Clinical Evaluation

A detailed medical history was collected from all the study participants. 

In the study group, the severity of skin lesions was estimated according to the modified Rodnan skin score, in which the degree of skin hardening is assessed with a scale of 0–3 points (0—no sclerosis, 1—slight sclerosis, 2—medium degree of induration, 3—high degree of induration) in 17 body areas (fingers, hands, forearms, arms, thighs, face, chest, feet). The total scale range is 0–51 [56].

Physical examination was performed on all study participants and included measurement of blood pressure and anthropometric parameters (BMI, waist, and hip circumference). In addition, the amount of adipose tissue using the bioimpedance method (BIA) (Tanita) was also assessed.

### 4.5. Blood Collection

Blood for laboratory tests was collected from all participants in standardized conditions after at least 6 hours of fastening. For the determination of adipokines, blood samples were collected in tubes containing EDTA, then centrifuged (3000 rpm) at 4 °C. The obtained plasma was stored at −80 °C.

Blood samples of systemic sclerosis individuals were obtained on the baseline and then after 6- and 9-month follow-ups.

### 4.6. Laboratory Examination

CRP, total cholesterol, triglycerides, low-density lipoprotein (LDL), high-density lipoprotein (HDL), and glucose were estimated using standard laboratory tests.

The concentration of omentin-1 in the blood plasma samples was determined using a commercial enzyme immunoassay ELISA kit (BioVendor, Brno, Czech Republic). The sensitivity of the test was 0.5 ng/mL. The inter- and intracoefficients of variations were <5%.

Total adiponectin and its high molecular weight fraction (HMW) in blood plasma samples were determined using a commercial enzyme immunoassay kit ELISA (ALPCO, Salem, NH). The sensitivity of the test was 0.034 ng/mL. The inter- and intracoefficients of variations were 4% and 5%, respectively.

The concentration of insulin in blood plasma samples was measured with a commercial RIA radioimmunoassay kit (DIAsource ImmunoAssays S.A., Louvain-la-Neuve, Belgium). The sensitivity of the kit was ≤1 µIU/mL. The inter- and intracoefficients of variations were 2% and 7%, respectively.

The insulin resistance was calculated based on the HOMA-IR index (calculated using the formula fasting glucose x fasting insulin/405, a value more than 2.5 was recognized as insulin resistance).

### 4.7. Statistical Analysis

The statistical analysis was performed in the R statistical package, version 4.0.5 (http://cran.r-project.org (accessed on 2 June 2021)). The significance level α = 0.05 was accepted as significant. The number of occurrences (*n*) and frequency (%) describe the nominal data. The normal distribution in individual subgroups was checked using the Shapiro–Wilk test. A comparison of the level of variance between subgroups was performed using Levene’s test. To determine whether there was a significant difference between the expected and observed frequencies in subgroups, the chi-square test was used.

The comparative analysis of the two subgroups was performed using the chi-square test or Fisher’s exact test for qualitative variables and for quantitative variables using the Student’s *t*-test for independent measurements or the Mann–Whitney U-test, as appropriate. When comparing the 3 subgroups, ANOVA was used with Tukey’s post-hoc test or Kruskal–Wallis test with Dunn’s post-hoc test with Bonferroni’s correction, depending on whether the assumptions were met. The analysis between individual measurements within the research group was performed. ANOVA for dependent measures or the Wilcoxon test for pairs was used in cases where the assumptions of ANOVA were not met. The relationship between the selected quantitative variables was verified using the Spearman correlation coefficient. In the research group, the correlation analysis was performed for each measurement separately due to the necessity to meet the assumption of the independence of observation.

## 5. Conclusions

Enhanced omentin levels were observed in patients with SSc as well higher levels of omentin-1 in patients with longer than 7 years disease duration may suggest that this adipokine is involved in the pathomechanisms of SSc as its concentrations are not directly related to BMI, age, and insulin resistance indices (HOMA-IR, fasting insulin levels). However, this hypothesis needs further evaluation in the larger patient cohort.

Our findings of the negative correlation between the concentration of adiponectin and the long-time duration of the disease, as well as the negative relation between the modified Rodnan skin score with the level of HMW adiponectin, strongly suggest that adiponectin is an important player in systemic sclerosis. However, intensive research is also needed here to clarify the exact role of adiponectin and its fraction in SSc.

## Figures and Tables

**Table 1:** Comparison of the anthropometric and biochemical parameters between the SSc and control group.

	SSc Group	Control Group	*p*
*n*	58	30	
Females. *n* (%)	46 (79.3)	25 (83.3)	ns
Age (years)	54.38 ± 13.42	49.57 ± 10.68	ns
BMI (kg/m^2^)	23.35 (21.39; 25.93)	24.71 (21.11; 27.70)	ns
HOMA-IR	2.03 (1.23; 3.60)	1.79 (1.41; 3.45)	ns
Insulin (µIU/mL)	8.56 (6.18; 16.04)	8.95 (6.42; 12.78)	ns
Waist (cm)	79.08 ± 10.41	83.04 ± 16.13	ns
Hip (cm)	97.94 ± 9.54	101.92 ± 9.95	ns
Fat mass (kg)	17.35 (11.30; 23.00)	20.10 (12.85; 24.05)	ns
FFM (kg)	44.00 (42.25; 47.95)	44.80 (42.25; 48.15)	ns
TBW (kg)	32.20 (30.95; 35.65)	33.30 (31.25; 36.60)	ns
SBP (mmHg)	118.05 ± 14.02	121.48 ± 8.08	ns
DBP (mmHg)	71.71 ± 8.66	71.90 ± 7.96	ns
Total cholesterol (mg/dL)	190.50 ± 42.80	194.53 ± 37.75	ns
HDL cholesterol (mg/dL)	62.48 ± 21.42	69.93 ± 17.45	ns
LDL cholesterol (mg/dL)	106.02 ± 38.14	107.67 ± 34.02	ns
TG Triglycerides (mg/dL)	90.50 (71.25; 127.50)	75.00 (55.25; 90.25)	0.024
Glucose (mg/dL)	86.00 (79.00; 96.00)	87.00 (82.00; 94.00)	ns
CRP (mg/L)	1.60 (0.80; 2.60)	0.63 (0.33; 2.29)	0.029

BMI (Body Mass Index); HOMA-IR (homeostatic model assessment for insulin resistance); FFM (Fat-Free Mass); TBW (Total Body Water) SBP (systolic blood pressure); DBP (diastolic blood pressure); HDL (high-density lipoprotein); LDL (low-density lipoprotein); TG (triglycerides); CRP (C-reactive protein). ns (non-significant). Data are presented as mean ± SD or median (Q1; Q3) in other cases.

**Table 2:** Comparison of adipokine concentrations in the group of patients with systemic sclerosis (study group) and the control group.

	SSc Group	Control Group	*p*
*n*	58	30	
Omentin-1 (ng/mL)	556.30 (428.12; 678.97)	486.37 (328.61; 555.67)	0.03
Total adiponectin (µg/mL)	8.20 (5.54; 10.34)	8.70 (5.54; 11.73)	ns
HMW Adiponectin (µg/mL)	2.90 (1.74; 4.46)	3.25 (1.71; 4.13)	ns

HDL—high-density lipoprotein. Data are presented as median (Q1; Q3).

**Table 3:** Correlations of adipokine concentrations with age and CRP levels.

Baseline	Omentin-1		Total Adiponectin		HMW Adiponectin	
	rho	*p*	rho	*p*	rho	*p*
Age	0.1	0.5	0.2	0.11	0.09	0.5
CRP	−0.09	0.48	−0.22	0.84	0.11	0.39

CRP—C-reactive protein.

**Table 4:** Comparison of the anthropometric and biochemical parameters and adipokines between the SSc subgroups and control group.

	Diffuse SSc	Limited SSc	Control Group	*p*	Post-Hoc
*n*	40	18	30		
Age (years)	54.00 (42.00; 63.00)	59.00 (50.75; 65.00)	51.00 (42.00; 55.00)	0.040	Limited SSc > control group
BMI (kg/m^2^)	23.25 (21.14; 25.59)	24.21 (21.81; 25.93)	24.71 (21.11; 27.70)	ns	
HOMA-IR	2.10 (1.27; 3.49)	1.63 (1.20; 3.60)	1.79 (1.41; 3.45)	ns	
Omentin-1 (ng/mL)	574.75 (449.74; 658.22)	522.42 (328.86; 680.16)	486.37 (328.61; 555.67)	ns	
Total adiponectin, (ug/mL)	7.93 (5.09; 10.48)	8.48 (6.05; 9.96)	8.70 (5.54; 11.73)	ns	
HMW-Adiponectin (ug/mL)	2.93 (1.63; 5.03)	2.78 (1.86; 3.69)	3.25 (1.71; 4.13)	ns	
Waist (cm)	76.00 (72.75; 86.25)	76.00 (74.00; 82.00)	77.50 (70.00; 95.00)	ns	
Hip (cm)	97.00 (91.00; 105.00)	99.00 (94.00; 100.00)	100.00 (95.25; 109.25)	ns	
Fat mass (kg)	15.70 (10.35; 22.95)	18.50 (12.70; 23.00)	20.10 (12.85; 24.05)	ns	
FFM (kg)	44.50 (42.40; 48.75)	43.90 (42.30; 47.00)	44.80 (42.25; 48.15)	ns	
TBW (kg)	32.60 (31.05; 36.50)	32.10 (31.00; 34.40)	33.30 (31.25; 36.60)	ns	
SBP (mmHg)	116.00 (109.25; 126.25)	122.50 (115.75; 129.75)	120.00 (115.00; 128.00)	ns	
DBP (mmHg)	70.00 (64.75; 80.00)	76.50 (70.00; 80.00)	72.00 (68.00; 78.00)	ns	
Total cholesterol (mg/dL)	173.00 (156.00; 204.00)	198.00 (176.00; 224.00)	186.00 (173.00; 211.75)	ns	
HDL cholesterol, (mg/dL)	63.00 (44.00; 80.00)	57.00 (49.00; 64.00)	67.00 (60.50; 79.75)	ns	
LDL cholesterol (mg/dL)	95.00 (76.00; 113.00)	115.00 (104.00; 141.00)	110.50 (79.25; 127.50)	ns	
TG (mg/dL)	86.00 (63.00; 128.00)	96.00 (81.00; 124.00)	75.00 (55.25; 90.25)	0.034	Limited SSc > control group
Glucose (mg/dL)	86.50 (80.75; 96.00)	81.00 (78.00; 93.00)	87.00 (82.00; 94.00)	ns	
CRP (mg/L)	1.70 (0.99; 2.51)	1.00 (0.60; 2.90)	0.63 (0.33; 2.29)	ns	

BMI (Body Mass Index); HOMA-IR (homeostatic model assessment for insulin resistance); HMW—high molecular weight; FFM (Fat-Free Mass); TBW (Total Body Water), SBP (systolic blood pressure); DBP (diastolic pressure); HDL (high-density lipoprotein); LDL (low-density lipoprotein); TG (triglycerides); CRP (C-reactive protein), ns (non-significant). Data are presented as median (Q1; Q3).

**Table 5:** Antibodies pattern in the SSc group.

	*n*	*n*%
Anti-Scl70	36	62.1
ACA	8	13.8
ANA *	3	5.2

* in patients with negative Anti-Scl70 or ACA. ACA (anti-centromere antibodies); ANA (anti-nuclear antibodies).

**Table 6:** Comparison of anthropometric parameters and adipokines between the SSc groups with different disease duration and the control group.

	SSc Group: Duration of the Disease <7 Years	SSc Group: Duration of the Disease ≥7 Years	Control Group	*p*	Post-Hoc
*n*	26	32	30		
Pulmonary involvement (*n*)	14	19			
Modified Rodnan skin score	6.00 (1.00; 8.00)	7.50 (5.00; 10.00)			
Age (yrs)	51.92 ± 14.17	56.38 ± 12.65	49.57 ± 10.68	ns	
BMI (kg/m^2^)	23.52 (20.97; 25.93)	22.87 (21.76; 25.33)	24.71 (21.11; 27.70)	ns	
HOMA-IR	1.63 (1.25; 3.32)	2.11 (1.23; 4.00)	1.79 (1.41; 3.45)	ns	
Omentin-1 (ng/mL)	517.35 (341.29; 610.23)	585.65 (453.38; 725.15)	486.37 (328.61; 555.67)	0.019	More than 7 years vs. control
Insulin (µIU/mL)	8.01 (6.17; 14.52)	9.42 (6.35; 16.48)	8.95 (6.42; 12.78)	ns	
Total adiponectin (µg/mL)	6.50 (4.60; 9.13)	8.93 (6.38; 10.98)	8.70 (5.54; 11.73)	ns	
HMW adiponectin (µg/mL)	3.25 (1.94; 5.45)	2.35 (1.58; 3.70)	3.25 (1.71; 4.13)	ns	
Waist (cm)	75.50 (73.00; 86.75)	76.00 (71.50; 84.50)	77.50 (70.00; 95.00)	ns	
Hip (cm)	100.00 (94.50; 105.50)	95.00 (93.00; 100.00)	100.00 (95.25; 109.25)	ns	
Fat mass (kg)	12.70 (10.70; 23.65)	17.50 (11.40; 21.80)	20.10 (12.85; 24.05)	ns	
FFM (kg)	44.90 (42.95; 47.70)	43.50 (41.60; 47.80)	44.80 (42.25; 48.15)	ns	
TBW (kg)	32.90 (31.45; 34.90)	31.80 (30.50; 36.40)	33.30 (31.25; 36.60)	ns	
CRP (mg/dL)	1.7 (0.60; 2.90)	1.56 (0.83; 2.43)	0.63 (0.33; 2.29)	ns	

BMI (Body Mass Index); HOMA-IR (homeostatic model assessment for insulin resistance); HMW (high molecular weight); FFM (Fat-Free Mass); TBW (Total Body Water), ns (non-significant). Data are presented as mean ± SD or median (Q1; Q3) in other cases.

**Table 7:** Correlations of adipokine concentrations with the modified Rodnan skin score.

Baseline	Omentin-1		Total Adiponectin		HMW Adiponectin	
	rho	*p*	rho	*p*	rho	*p*
Rodnan Scale	−0.05	0.689	−0.24	0.077	−0.27	0.048

HMW—high molecular weight.

**Table 8:** Comparative analysis of selected anthropometric parameters, the modified Rodnan skin score, and adipokine concentrations in patients with systemic sclerosis in a 6-month and 9-month follow-up.

	Baseline	6-Month Follow-Up	9-Month Follow-Up	*p*
BMI (kg/m^2^)	23.79 ± 3.71	23.98 ± 3.67	24.05 ± 3.72	ns
Omentin-1 (ng/mL)	582.57 (453.92; 694.72)	553.53 (411.97; 705.22)	549.24 (423.47; 713.89)	ns
Total Adiponectin, (µg/mL)	8.30 (5.65; 10.00)	7.50 (5.95; 9.80)	6.85 (5.65; 9.90)	ns
HMW Adiponectin, (µg/mL)	3.05 (1.78; 3.95)	2.45 (1.40; 3.73)	2.15 (1.35; 3.35)	ns
Waist (cm)	75.50 (72.75; 84.00)	78.50 (72.00; 85.00)	78.50 (71.75; 85.00)	ns
Hip (cm)	98.44 ± 10.05	98.53 ± 9.50	98.91 ± 9.59	ns
Fat mass (kg)	17.80 (12.18; 22.70)	16.25 (6.10; 40.00)	15.70 (11.13; 21.70)	ns
FFM (kg)	43.70 (42.38; 45.08)	44.35 (40.60; 55.30)	43.80 (42.40; 48.38)	ns
TBW (kg)	31.95 (31.00; 36.15)	32.50 (29.70; 40.50)	32.10 (31.00; 35.40)	ns
SBP (mmHg)	116.62 ± 12.92	119.73 ± 16.63	117.65 ± 14.07	ns
DBP (mmHg)	70.58 ± 9.49	70.69 ± 8.09	71.77 ± 6.80	ns
Total Cholesterol (mg/dL)	177.47 ± 41.09	182.37 ± 41.51	184.37 ± 34.64	ns
HDL (mg/dL)	58.47 ± 18.21	59.53 ± 20.07	54.79 ± 20.13	ns
LDL (mg/dL)	96.53 ± 40.25	97.37 ± 37.27	103.21 ± 35.57	ns
TG (mg/dL)	90.00 (70.00; 121.00)	124.00 (51.00; 307.00)	110.00 (102.00; 137.00)	ns
Insulin (µIU/mL)	8.24 (6.12; 16.48)	7.59 (5.47; 15.76)	8.03 (6.14; 21.49)	ns
Glucose (mg/dL)	85.00 (78.00; 96.00)	86.50 (68.00; 154.00)	87.50 (80.00; 94.50)	ns
Modified Rodnan Skin Score	7.50 (5.00; 10.00)	7.00 (0.00; 16.00)	7.50 (4.00; 10.00)	ns

BMI—body mass index; HMW—high molecular weight; FFM—Fat-Free Mass; TBW—Total Body Water; SBP—systolic blood pressure; DBP—diastolic pressure; HDL—high-density lipoprotein; LDL—low-density lipoprotein; TG—triglycerides; CRP—C-reactive protein; ns—non-significant. Data are presented as mean ± SD1 or median (Q1; Q3) in other cases.

**Table 9:** Comparison of comorbidities between the SSc and control group.

	Study Group (*n* = 58)	Control Group (*n* = 30)	RR (95% CI)	*p*
Diabetes type 2	6 (10.3)	1 (3.3)	3.10 (0.39; 24.61)	ns
Coronary heart disease	5 (8.6)	0 (0.0)	-	ns
Myocardial infarction	1 (1.7)	0 (0.0)	-	ns
Hypertension	22 (37.9)	4 (13.3)	2.84 (1.08; 7.50)	0.025

Data are presented as *n* (% of the group). ns—non-significant. Groups were compared using Fisher’s exact test. RR—risk ratio for the research group concerning the control group.

## Data Availability

The data presented in this study are available on request from the corresponding author. The data are not publicly available due to privacy restrictions.

## List of Abbreviations

ACA	anti-centromere antibodies
AMPK	5′ AMP-activated protein kinase
ANA	anti-nuclear antibodies
BIA	bioimpedance assessment
BMI	Body Mass Index
COX-2	cyclooxygenase-2
CRP	C-reactive protein
eNOS	endothelial nitric oxide synthase
HDL	high-density lipoprotein
HMW	high molecular weight
HOMA-IR	homeostatic model assessment of insulin resistance
ICAM-1	intercellular adhesion molecule-1
IL-1	interleukin-1
IL-6	interleukin-6
IR	insulin resistance
LDL	low-density lipoprotein
LMW	low molecular weight
MMW	medium molecular weight
MAPK	mitogen-activated protein kinase
NF-κB	nuclear factor kappa-light-chain-enhancer of activated B cells
NO	nitric oxide
PPARγ	peroxisome proliferator-activated receptor gamma
ROS	reactive oxygen species
RVSP	right ventricle systolic pressure
SSc	systemic sclerosis
TGF-β	transforming growth factor β
Th	T helper lymphocyte
TNFα	tumor necrosis factor α
VAT	visceral adipose tissue
VCAM-1	vascular adhesion molecule-1
